# Substandard Quality of the Antimicrobials Sold in the Street Markets in Haiti

**DOI:** 10.3390/antibiotics9070407

**Published:** 2020-07-14

**Authors:** Théodule Jean-Baptiste, John F. Carpenter, Kevin Dahl, Wellington Derameau, Rosemela Veillard, John Redford Jacquet, Pierre Ludens Osselyn, Albert Figueras

**Affiliations:** 1 LABMES, Faculté de Médecine et de Pharmacie, Université d’Etat d’Haïti, Port-au-Prince HT6110, Haiti; dwellington94@gmail.com (W.D.); jacquetjephney@gmail.com (J.R.J.); osselynludens21@gmail.com (P.L.O.); 2 Groupe Haitien de Recherche, d’Innovation et de Créativité (GHRIC), Tabarre HT6124, Haiti; veillardrosemela14@gmail.com; 3 Department of Pharmaceutical Sciences, University of Colorado Anschutz Medical Campus, Aurora, CO 80045, USA; JOHN.CARPENTER@cuanschutz.edu; 4 KBI Biopharma, Boulder, CO 80301, USA; kdahl@kbibiopharma.com; 5 Departament de Farmacologia, Terapèutica i Toxicologia, Universitat Autònoma de Barcelona, E-08035 Barcelona, Spain; albert.figueras@gmail.com

**Keywords:** pharmaceutical quality, antimicrobial resistance, substandard medicines, Haiti, street markets, AWaRe classification

## Abstract

This pilot study was conducted to analyze the quality of the antimicrobials sold in the street markets in Port-au-Prince, Haiti. A total of 258 packs containing antimicrobials were bought in 28 street markets in Port-au-Prince (Haiti). Tablets and contents of capsules included in 196 packs were analyzed using a Raman handheld spectrometer (NanoRAM of BWTEK, Model: BWS456-785) during the first quarter of 2019. Three out of 11 antimicrobials (Amoxicillin, Metronidazole, and Cotrimoxazole) had a high spectral match with an HQI ≥ 90 to the respective authentic medicine for more than 95% of their tablets/capsules. For six antimicrobials (Tetracycline, Erythromycin, Cloxacillin, Azithromycin, Clarithromycin, and the combination Amoxicillin + Clavulanic Acid) none of their tablets/capsules showed a sufficient spectral match with the authentic medicine. This finding indicates that these products sold in the markets did not contain the labeled drug and/or contained a degraded drug. In addition to the fact that prescription antimicrobials can be purchased in street markets, the present field study found that for most of them (including “Watch” antimicrobials according to the AWaRe classification) were substandard, which contributes to the present antimicrobials resistance epidemic.

## 1. Introduction

According to the World Health Organization (WHO) Global Surveillance and Monitoring System, an estimated 1 in 10 medical products circulating in low- and middle-income countries are either substandard or counterfeit [1,2]. The WHO estimates that around 30% of the medicines sold in countries of Asia, Africa, and Latin America are substandard or counterfeit [3]. And many times, this situation can have enormous consequences for public health; for example, up to 64% of antimalarial drugs in Nigeria were found to be counterfeit in 2011 [4]. Worldwide, around 10% of all medicines could be substandard or counterfeit [5]. The counterfeit problem is especially acute in low- and middle-income countries (LMICs) as compared to high-income countries. However, the availability of medicines through the Internet has also expanded the problem to high-income countries. In general, counterfeiting involves both lifestyle and lifesaving medicines, including antimicrobials, because counterfeiting pharmaceutical product is hugely profitable, and current penalties are insufficient to deter this practice [6].

Antimicrobials are medicines especially relevant because, after their golden age lasting more than 40 years in the 20th Century, the growing evidence on resistant strains to old and new antibiotic molecules became a global threat [7]. A simulation made by the World Bank suggested that antimicrobial resistance (AMR) could have a devastating impact on millions of people as well as destructive impacts on the global economy from 2017 through 2050 [8]. According to that report, in a high AMR-impact scenario, the world would lose 3.8 per cent of its annual gross domestic product (GDP) by 2050, a problem which has a more significant impact on LMICs [8].

One of the most important causes of AMR is an inappropriate use of antibiotics, either at inadequate dosages or duration or its consumption for non-bacterial diseases [9,10,11], not only by humans but also its use and presence in livestock or the environment [12,13]. This knowledge leads to the *One Health* approach to try to reverse the present tendency [14]. It is also well-known that the vast majority of human antimicrobials use occurs in the community, where such drugs can readily be obtained, even without a prescription [15,16,17]. Some of these products containing antimicrobials are stored for self-medication use, also to treat conditions not always requiring an antibiotic; this is also a widespread phenomenon [18,19].

Antibiotics and antimalarials are the most commonly counterfeit medicines [20]; additionally, street markets selling medicines are common practice in many LMICs. Thus, we designed the present pilot study with the main purpose of analyzing the quality of the antimicrobials sold in the street markets in Port-au-Prince, Haiti.

## 2. Results

A total of 258 packs of antimicrobials containing 21 generic medicine names labelled on the package were bought from sellers in 28 public markets; 196 packs including 11 antimicrobials were analyzed.

### 2.1. Medicines Analyzed

Amoxicillin was the most tested antimicrobial (58 packs), followed by metronidazole (42 packs) and cotrimoxazole (28 packs) (Table 1). The manufacturing date of the medicine was absent on 55 packs (28.1%), and five packs (2.6%) did not have an expiration date on the box. The majority of the medicines analyzed (147 packs; 75.0%) came from India or China (14; 7.1%). The other countries of origin were Kenya (11; 5.6%), United States of America (10; 5.1%), Haiti (3; 1.5%), France (1; 0.5%), Canada (1; 0.5%), and Honduras (1; 0.5%). Eight packs (4.1%) did not note a country of origin.

### 2.2. Spectrum Quality

Three out of 11 antimicrobials (Amoxicillin, Metronidazole, and Cotrimoxazole) had a good spectral match for more than 95% of their tablets/capsule. However, for 6 antimicrobials (Tetracycline, Erythromycin, Cloxacillin, Azithromycin, Clarithromycin and the combination Amoxicillin + Clavulanic Acid) none of their tablets/capsule showed a sufficient spectral match with the authentic medicine. Finally, Ciprofloxacin and Chloroquine had 63% and 89% of their tablets/capsule with good spectral match (Table 2). The proportion of poor spectral match among the analyzed “Watch” antimicrobials was 61% of 130 samples, while the poor spectral match among the analyzed “Access” antimicrobials was 16.9% of 765 samples.

### 2.3. Quality of Spectrum within a Pack

Another interesting aspect studied was the potential quality differences within a single pack. Our spectrum analyses showed that for two products (amoxicillin and chloroquine) there was heterogeneity in the spectra of the units within a given pack. For amoxicillin, two packs had each one unit out five with poor spectrum and another pack had two units with poor quality spectra. Four packs of chloroquine had one unit out of five units analyzed with poor quality spectrum. For the other antimicrobials, the spectrum quality was homogenous within a pack.

## 3. Discussion

The main result of this study of the quality of some antimicrobials sold in street markets of Haiti showed that antimicrobials could be freely obtained without a prescription, and many of the antimicrobials were products of particular interest (“Watch” category). For example, 15 packs of 9 different antimicrobial molecules were bought at the public market in Croix des Bouquets and 13 packs of 8 molecules in “Marché Telele”. Further to this, more than half of the analyzed samples had poor quality based on the lack of sufficient Raman spectral match with reference products. Thus, the presence of antimicrobials in street markets, which facilitates an irrational use, and their poor pharmaceutical quality are factors which contribute to antimicrobial resistance. Additionally, in a few cases, differences in the quality of units within the same pack were identified, a heterogeneity which should be taken into account by policy makers to design technical inspections.

Moreover, the Hit Quality Index (HQI) only tells the correlation between spectra for authentic medicine and the purchased antimicrobial. Still, it does not assess the quality of the active ingredient. Nor are the compounds present in counterfeit medicines necessarily identified. Thus, degraded and/or mislabeled drugs are likely sold in the street markets, further contributing to antimicrobial resistance.

One of the major appeals of medicines sold in street markets for the client is the cost and the time saved by going straight to the seller, compared to the amount of time and money spent in formal health facilities [21].

Although these substandard, counterfeit, and falsified medicines can be found everywhere, they are more frequent in places with informal markets where prescription medicines are freely sold by street sellers, and buyers can acquire different kinds of products at will [22]. In 2014, a pilot study of market surveillance was performed in Senegal; the authors analyzed the best selling drugs from an official pharmacy and a street market in two principal cities of the country and some traditional preparations from herbal medicine from the same market [23]. They found that four best-selling products purchased from a Dakar local pharmacy exactly contained the amount of active principles reported in the respective labels. In contrast, the top-selling medicines purchased from Kaolack market contained an amount of active ingredients lower than that declared on the label. Notwithstanding this, none of the analyzed products was an antimicrobial.

Another study conducted in Dande, Bengo Province, Angola, analyzed the problem from a different perspective. It was a cross-sectional pilot study, including 102 households within the 10th Health and Demographic Surveillance System round [24]. Its conclusions highlighted that more than 66% of the antimicrobials stored had been prescribed by a health professional and the majority of antimicrobials were bought at pharmacies or a street market, thus the dynamics of street markets in different countries, as well as the pharmaceutical quality associated with these products freely sold. Related with the over-the-counter access to antimicrobials, a recent study conducted in Tanzania found that 13 out of 14 community pharmacies offered over-the-counter antibiotics for upper respiratory symptoms [25].

To our knowledge, this is the first study approaching the free sales of antimicrobials in Haiti using this technique. The NanoRAM portable Raman spectrometer is a relatively new technology using a non-destructive and accurate approach to the detection of counterfeit pharmaceutical products [26,27,28].

Despite potential limitations such as the sampling process, the main objective of this pilot study was to show the feasibility of conducting such analyses in countries as Haiti and highlighting the relevance and potential consequences of the problem of unregulated sales of antimicrobials by street vendors.

## 4. Materials and Methods

We identified and selected 28 frequented public markets in the eight communes of Port-au-Prince (Haiti). Four research assistants bought different brands of antimicrobials products as “mystery shoppers” in these markets between January and April 2019. There was no randomization in the markets and the sellers’ selection process; the shoppers bought the medicines from any seller that had antimicrobials available for sale.

### 4.1. Quality Testing

The medicines bought were analyzed using a Raman handheld spectrometer (NanoRAM of BWTEK, Model: BWS456-785, Serial Number: SAGFMA, manufactured on March 2013) [26], which assesses the quality of a given component by acquiring its spectrum and comparing it for similarity with a database containing predefined spectra of authentic medicine molecules. The result of the comparison is given either using a probabilistic approach expressed as a p-value or a correlation algorithm between the tested component and library reference spectra expressed as Hit Quality Index (HQI) [26,27,28]. The value of using the Raman spectroscopy lies in the fact that each medicine has a specific and unique spectrum that is often referred as a “fingerprint” [29]. In our study, a HQI greater or equal to 90 was considered to be a “good spectral match” between the analyzed units and their authentic counterpart [27].

The NanoRAM sensitivity and specificity in identifying medicines has been documented and reported in several studies supporting its validity and reliability for field study [29,30]. The NanoRAM is a recognized instrument in compliance with the requirements of the Pharmacopeia in the US, Europe, and China [29]. The NanoRAM comes with more than 100 built-in spectra. This spectra library was expanded in University of Colorado Denver by adding extra spectra of antimicrobials, namely “Methods” in the language of the manufacturer. Although Raman spectrometer is a technology that requires minimal training [29,31], the research team was trained on its use in Denver School of Pharmacy on November 2018.

For the analyses, the tablets were placed in the tablet holder, and the capsule powders were poured into sample vials. For each pack of medicines, assuming a certain level of homogeneity within the pack, we analyzed half of the content of pack, which was five units most of the time as the majority of the antimicrobials bought came in a pack of 10 units except for two packs of azithromycin that had respectively 3 and 2 units of which all the units were analyzed. The remaining pills were kept for another study. The match or no match comparison decisions were performed by the software built into the NanoRam based on the HQI settings described above. Data were recorded in a Microsoft Excel file. Data analysis was done using the R statistical software, version 3.6.1.

### 4.2. Classification of the Antimicrobials

To try to accelerate the fight against antimicrobial resistance, the WHO Expert Committee of Essential Medicines proposed a key policy tool known as the AWaRe classification of antibiotics in 2017 [32]. The AWaRe classification categorizes antimicrobials in three groups: “Access”, “Watch”, and “Reserve”, where Access antimicrobials are first or second-line treatments for common infections and should be widely accessible. Antimicrobials classified in the Watch category should be prescribed only to a limited group of well-defined infections since they are at higher risk of bacterial resistance. Lastly, antimicrobials classified as Reserve are to be used for the treatment of infections by microorganisms resistant to multiple antibiotics [33,34]. Thus, AWaRe was used for the categorization of the antibiotics found in the analyzed products obtained in street markets.

## 5. Conclusions

In conclusion, a proportion of the antimicrobials sold in street markets in Haiti had poor pharmaceutical quality or are counterfeit. The number of substandard products seems higher among quinolones and macrolides, “Watch” antimicrobials, which consumption and use should be specially surveilled.

## Figures and Tables

**Table 1:** Description of the 196 packs and 975 units acquired in the selected street markets of Port-au-Prince (Haiti) used for the analyses conducted in the present study (see Methods). The table also includes the price range (in US$) of the different products and the countries of origin of each pack.

Generic Name	Number of Packs	Number of Units	Price Range * for a Pack in US$ **	Countries of Origin
Amoxicillin	58	290	0.5–0.76	India (50), China (4), Haiti (1), Not available (3)
Metronidazole	42	210	0.5–1.51	India (40), China (1), Honduras (1)
Cotrimoxazole	28	140	0.5–6.97	Kenya (11), China (9), India (7), Not available (1)
Tetracycline	21	105	0.5–0.61	India (16), USA (5)
Chloroquine	16	80	0.5–1.00	India (15), France (1)
Ciprofloxacin	16	80	0.5–2.78	India (9), USA (5), Haiti (1), Canada (1)
Erythromycin	7	35	0.76–1.82	India (4), Not available (3)
Azithromycin	3	10	0.5–0.76	India (2), Not available (1)
Cloxacillin	3	15	1.26–1.7	India (2), Haiti (1)
Amoxicillin/Clavulanic Acid	1	5	1.00	India (1)
Clarithromycin	1	5	4.04	India (1)

* minimum—maximum price; ** 100 Haitian gourdes = 1 US$ (February 2019).

**Table 2:** Description of the spectral match quality (“Good” or “Poor”) of the analyzed sample of antimicrobials acquired in different street markets in Port-au-Prince (Haiti). The different medicines have been categorized according to the AWaRe classification (see Methods).

AWaRe Classification	Generic Name	Good Match	Poor Match	Total Samples
		n (%)	n (%)	n (%)
Access	Amoxicillin	286 (98.6)	4 (1.4)	290 (100)
	Amoxicillin/Clavulanic Acid	-	5 (100)	5 (100)
	Cloxacillin	-	15 (100)	15 (100)
	Cotrimoxazole	140 (100)	-	140 (100)
	Metronidazole	210 (100)	-	210 (100)
	Tetracycline	-	105 (100)	105 (100)
Subtotal Access		636 (83.1)	129 (16.9)	765 (100)
Watch	Azithromycin	-	10 (100)	10 (100)
	Ciprofloxacin	50 (62.5)	30 (37.5)	80 (100)
	Clarithromycin	-	5 (100)	5 (100)
	Erythromycin	-	35 (100)	35 (100)
Subtotal Watch		50 (38.4)	80 (61.5)	130 (100)
–	Chloroquine	71 (88.8)	9 (11.3)	80 (100)
TOTAL		757 (77.6)	218 (22.4)	975 (100)
